# Construction of educational technology on non-violent communication between health professionals: an experience report

**DOI:** 10.1590/0034-7167-2022-0414

**Published:** 2023-04-14

**Authors:** Paula Arquioli Adriani, Paula Hino, Mônica Taminato, Hugo Fernandes

**Affiliations:** I Universidade Federal de São Paulo. São Paulo, São Paulo, Brazil

**Keywords:** Educational Technology, Health Communication, Interpersonal Relations, Health Personnel, Workplace Violence, Tecnología Educacional, Comunicación en Salud, Relaciones Interpersonales, Personal de Salud, Violencia Laboral, Tecnologia Educacional, Comunicação em Saúde, Relações Interpessoais, Pessoal de Saúde, Violência no Trabalho

## Abstract

**Objective:**

to report the construction of an educational technology to promote non-violent communication for health professionals.

**Methods:**

an experience report on the development of an educational technology on non-violent communication for health professionals, prepared by members of a social university extension project. The Plan-Do-Study-Act cycle was used as a process or product management procedure.

**Results:**

two complete management method cycles were performed. A mini almanac was generated as a final product, which addressed the main elements of non-violent communication, an example of its use in everyday life, hobbies and interspersed activities.

**Conclusion:**

educational technology construction (mini almanac) by members of a university extension project was facilitated using the Plan-Do-Study-Act cycle, proving to be a resource for disseminating non-violent communication in health work and promoting a culture of peace.

## RESUMO

**Objetivo:**

relatar a construção de uma tecnologia educacional de promoção da comunicação não violenta para profissionais de saúde.

**Métodos:**

relato de experiência sobre o desenvolvimento de uma tecnologia educacional de comunicação não violenta para profissionais de saúde, elaborada por membros de um projeto social de extensão universitária. Foi utilizado o ciclo *Plan-Do-Study-Act* como procedimento de gestão de processo ou produto.

**Resultados:**

foram executados dois ciclos completos do método de gestão. Foi gerado como produto final um mini almanaque, que abordou os elementos principais da comunicação não violenta, exemplo de seu uso no cotidiano, passatempos e atividades intercaladas.

**Conclusão:**

a construção da tecnologia educacional (mini almanaque) por membros de um projeto de extensão universitária foi facilitada com uso do ciclo *Plan-Do-Study-Act*, mostrando-se um recurso de difusão da comunicação não violenta no trabalho em saúde e promoção da cultura de paz.

## RESUMEN

**Objetivo:**

relatar la construcción de una tecnología educativa para promover la comunicación no violenta para los profesionales de la salud.

**Métodos:**

relato de experiencia sobre el desarrollo de una tecnología educativa de comunicación no violenta para profesionales de la salud, elaborado por integrantes de un proyecto de extensión universitaria social. Se utilizó el ciclo Plan-Do-Study-Act como procedimiento de gestión de procesos o productos.

**Resultados:**

se realizaron dos ciclos completos del método de manejo. Se generó como producto final un mini almanaque, que abordó los principales elementos de la comunicación no violenta, ejemplo de su uso en la vida cotidiana, aficiones y actividades intercaladas.

**Conclusión:**

se facilitó la construcción de tecnología educativa (mini almanaque) por integrantes de un proyecto de extensión universitaria utilizando el ciclo Plan-Do-Study-Act, demostrando ser un recurso para la difusión de la comunicación no violenta en salud y promoción del trabajo de la cultura de paz.

## INTRODUCTION

Interpersonal relationships at work can be understood as interactions between professionals from the same institution or service, favoring the harmonious achievement of common goals. It is expected that coexistence is as pleasant as possible and promotes well-being during work activities. For this, workers constantly resort to communication as a relationship tool. The use of communication can both favor healthy interpersonal relationships between health professionals and can be harmful, leading to conflicts, intensifying problems and discomforts that can affect the health of those involved, in addition to reducing the achievement of common goals^(1-2)^.

Communication at work can take many forms, usually being described as internal and interpersonal. Internal communication at work can be understood as a set of actions used by organizations and services that enable the relationship with the internal public so that messages can be captured by employees, understood and used in order to promote actions by companies or their managers. Formal language (such as writing) almost always prevails to convey information in a way that facilitates the company-worker relationship. In this type of communication, transmission is often unidirectional, planned by managers and with little participation of those who execute the outlined ideas^(2-3)^. Even the little exchange between those involved causes many proposals to fail, as workers do not always understand the information or have space for dialogue with decision-makers.

Interpersonal communication, on the other hand, is more delicate, it involves the exchange of information between two or more subjects, in a relationship that is not necessarily always focused on work or for the direct achievement of results. It is guided in different ways, with extensive use of verbal and non-verbal signals, creating denser and more meaningful interactions for those involved so that it often awakens affections or disaffections that transcend the space and time of work^(1,4)^.

Researchers from different areas of knowledge have focused on interpersonal communication, in order to better understand points such as: adaptations and changes in verbal and non-verbal communication, especially in the era of technological communication channels, such as social networks; ways of producing and interpreting messages or signals and how this influences the behaviors of those involved; and art of dialogue, or relational dialectic^(1-5)^.

Non-violent communication (NVC) emerged as a light technology based precisely on the certainty that conflicts cannot always be eliminated or avoided. On the contrary, they are fundamental for reflections and growth of all those involved. However, the proposal is to make use of resources that reject violence (in its different forms) as a way of solving problems. Therefore, its widespread and frequent use can benefit both home and work environments^(6)^. Adequately planned health education actions can benefit from NVC, given that its implementation process favors the careful observation of reality, can help identify feelings, sometimes not clearly reported, and encourage meeting participants’ needs through clear and directive requests. NVC scholars point out that it can be intimately collaborative with educational technologies, as it facilitates or mediates teaching and learning processes, in an innovative and pleasant way, transcending the operationalization of actions foreseen in pedagogical planning, to promote more lasting and significant cultural transformations^(5)^.

Studies on using interpersonal communication in health work have been frequent and explore important points^(1-5)^, but most are focused more on the potential repercussions for service users than for the workers themselves. An exception was found in a multicenter study that sought to identify the level of interpersonal communication competence among nursing students and to correlate their domains with some sociodemographic variables^(1)^. Among the findings, the influence of participation in extension projects stands out as a potentiator for a more assertive and positive interpersonal communication to subjects.

In this aspect, university extension constitutes one of the tripods of academic training and allows the return of knowledge to society, in addition to allowing the dialogic exchange between the external community and the university, favoring the construction of a more just, democratic and ethical society. The Brazilian National University Extension Policy (*Política Nacional de Extensão Universitária*) provides that extension should be a protagonist in bringing together the population’s needs and the academy so that the latter can produce science and teach according to the imperatives of collectivity^(7)^. The document itself highlights some thematic areas, which should guide the systematization of actions in major areas of social interest, namely: culture, human rights and justice, education, health, technology, work, and communication. In other words, the communication theme is also relevant as a thematic axis for extension actions, with potential merit for minimizing the problems and challenges of society.

One of the possibilities of university extension is through social projects and programs, understood as procedural and continuous actions of a social, cultural, technological or scientific nature, with clear and well-founded objectives that involve society and the university^(7)^. There are countless potentialities in social projects and programs, such as the creation of technologies (soft, soft-hard or hard). Extension projects aimed directly or indirectly at health professionals can make use of these technologies, such as NVC, as their operation is quite plausible and pleasant. However, it requires continuous application and daily exercise so that its concepts are actually used, reducing friction caused by alienating communication. Thus, creating educational products that encourage and favor the exercise of NVC can be very opportune to improve the interpersonal relationships of health professionals.

Given these aspects, the intimate relationship between communication and interpersonal relationships in health, NVC as a potential method of conflict resolution and the multiple paths that university extension has, a proposal to create an educational technology for NVC promotion among health professionals in general emerged.

## OBJECTIVE

To report the construction of an educational technology to promote NVC for health professionals by members of a social university extension project.

## METHODS

This is an experience report on the construction of an educational technology to promote NVC for health professionals, prepared by members (undergraduate nursing students, graduate students, professors, health professionals and community representatives) of a social university extension project called “*Jano - Cultura de Paz*”, between July and December 2020.

The aforementioned project aimed to articulate and implement university extension actions aimed at achieving the premises of a culture of peace established by the United Nations Educational, Scientific and Cultural Organization (UNESCO), namely: respect for life, rejection of violence, use of generosity, listening for understanding, preservation of the planet and rediscovery of solidarity. According to UNESCO, people, families, communities and even countries must make use of these premises in their daily lives, in order to allow for the expansion of collective well-being, security and the feeling of peace among all^(8)^. Such premises are not utopian and can be achieved through behavioral changes, sometimes simple. *Jano* project activities have been taking place since 2018 at *Universidade Federal de São Paulo* (UNIFESP), São Paulo campus, with the coordination of two professors and the participation of undergraduate and graduate students, professionals and members of the community surrounding the campus. Their meetings usually take place every fortnight, in person or virtually. All members are invited to reflect on premises established by UNESCO as horizontally as possible.

In 2020, the UNIFESP nursing course began its first experiences in crediting extension hours in undergraduate curricular units, as provided for in goal 12.7 of the Brazilian National Education Plan, which allowed all students to experience university extension more intensely during their training. Thus, *Jano* project started its participation in the Education, Communication and Health curricular unit, contributing especially in the communication axis. The educational technology of this report was elaborated from this initial process of approaching the extension of the curricular activities foreseen in the nursing course’s pedagogical project.

The product, the result of this report, was awakened with a brainstorming technique, and the elected proposals were instrumentalized through the interactive process and product management method called PDSA cycle^(9)^, whose acronym is related to verbs To Plan, To Do, To Study and To Act.

Initially, eight extension workers, a fellow extension monitor, two professors, an active health professional and two representatives from the external community met in July 2020 to discuss the prospects for *Jano* project accreditation in the Education, Communication and Health curricular unit. Professors sought to act as facilitators of the discussion, raising the following question: how to promote, through university extension, communication based on a culture of peace among health professionals? Thenceforth, the brainstorming technique began, through which the facilitators explored the skills and creativity of the group. For this, the following steps were taken: 1) setting the time limit of 60 minutes for dynamics; 2) presenting concise information on the topic, target population and potential extension actions; 3) encouraging attentive listening and devoid of prior judgment; 4) encouraging the presentation of ideas, even the most unusual or strange; 5) encouraging the group to choose the best ideas; and 6) screening to find the most appropriate idea for the group. At the end of this process, the members chose the construction of a mini almanac for health professionals, having NVC as a theoretical-methodological framework.

Thus, the PDSA cycle was started, taking two complete cycles to build the desired product, here divided into phases 1 and 2.

## RESULTS

In phase 1, To Plan step, members planned a schedule of meetings and goals for each meeting, whose final product would be presented in a maximum of six months. The necessary tools and programs or applications for developing the proposal were also chosen. Members have also been divided into tasks to facilitate the start of the process. The next step, To Do, involved familiarization with selected applications and construction of an educational pre-project. The To Study step, on the other hand, included an in-depth study of possible educational technologies and reflection on the results obtained in the previous step, promoting adaptations in planning. It was in this step that extensionists found in the scientific literature educational almanac as a potential technological resource to meet the proposed objective, choosing it as a strategy to be adopted. Its choice was justified by allowing the use of proactive activities that would overcome the model of transmission by reading, such as a booklet. In To Act step, the following actions of a new improvement plan were carried out: a) establishment of activity standards for the educational product (mini almanac), such as crosswords, connecting dots, texts and answering questions; and b) construction of a prototype, with free hand drawings, in sulfite sheets.

**Figure 1:**
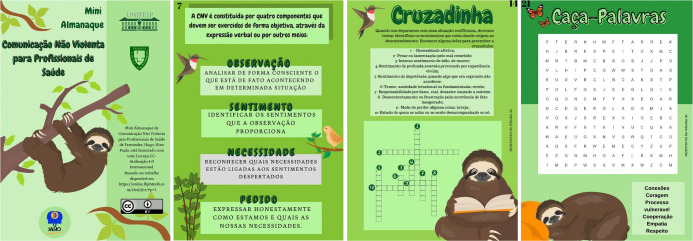
Partial illustrative representation of the final version of the mini almanac, São Paulo, São Paulo, Brazil, 2021

During phase 2, the same steps were taken with replanning the format, expanding the content and executing the activities. In To Plan step, the extension workers planned their distribution in tasks for elaborating the mini almanac activities. In To Do step, they executed the action plan, building the mini almanac pages in free image and text applications chosen on the internet so that the sketches traced in the prototype would materialize. In To Study step, members revisited the literature to insert more precise concepts, bringing the origins of culture of peace in search of transcendence of the NVC technique, in addition to inserting a practical example of its use. They also reflected on the playfulness of images and texts so that there would be entertainment and avoid fatigue during the actions. Finally, in To Act step, the materials produced were sorted and transported to another application, which converted the selection into a single file, with the effect or sensation of “flying through” the pages, obtaining the final product called “*Mini Almanaque Comunicação Não Violenta para Profissionais de Saúde*”, as illustrated in Figure 1.

The total number of people involved was 14, including two professors, one being a researcher specializing in health communication, two graduate students, two community members (health professionals) and eight second-year undergraduate nursing students. It took six meetings, from planning to product presentation. The final product was presented to people outside the project to identify interest, compliance and familiarity with the activities provided for in the mini almanac. The educational technology created proved to be very attractive, playful and easy to understand, allowing wide use among health professionals of different levels of education.

It is noteworthy that the images adopted were already images of the application used, with permission to use and share for free. Thus, no new images were created. Choosing the sloth as a mascot for this mini almanac was due to being an animal that symbolizes wisdom and serenity, subliminal essences propagated by NVC.

To guarantee product legitimacy, as well as to allow its wide use, the authors registered a license with the Creative Commons International 4.0 organization, with public attribution and free use. As a way of guaranteeing the preservation of intellectual production, the material was deposited in the Institutional Repository of UNIFESP, under number 60428, available at electronic address https://repositorio.unifesp.br/handle/11600/60428.

## DISCUSSION

Conflicts in health work environments can have different origins, but the proper use of communication can facilitate interaction between people and help in solving problems that affect those involved, witnesses and even the institution^(3-4)^. Thus, NVC is feasible and timely for everyday use by healthcare professionals. However, as it is a relatively new technique and little explored in the field of work, there is a possibility that few people know about it and practice it successfully.

Playful technologies, such as the one adopted in this report, allow dense or very technical information to be socialized in a more pleasant and fun way, making the learning process easier, bringing abstract concepts closer to everyday practices. This means that learners do not necessarily notice that they are being instructed, but end up learning information that will be reflected in their attitudes^(10)^.

The use of almanacs for health education has been increasing, especially in nursing, with successful measures among different populations of health service users. Its use is also potential for continuing education of workers, as it constitutes a leisure activity, but educational, to professionals who often have little time or little opportunity to take courses, workshops or similar^(10)^. In this regard, a mini almanac aimed at promoting the use of NVC can bring the theme closer to health professionals in a subtle way, with few negative impacts on daily lives and work demands of these people.

It is noteworthy that NVC is not a technology that preaches meekness, modest or excessively flexible behaviors in order to avoid discord. It is a process that involves the use of four key elements or components that allow for honest, empathetic and assertive expression^(6)^. These components are: (1) observation, which is the conscious analysis of the facts that happen in a situation; (2) identification and expression of feelings, which seeks to identify feelings in oneself or in others through observation; (3) recognition of needs, which are essential particularities that need to be met; and (4) request, which is a clear and honest request for action that can assist in resolving the conflict^(6,8)^.

Learning this technique and its routine use can not only favor healthier or more pleasant work environments, but can positively affect practitioners’ quality of life and sense of well-being, proving to be an important tool for a culture of peace^(6,8)^. Associated with this, the construction of an educational technology in a university extension project made it possible for students and extension workers to share the knowledge acquired with the community, as well as having it as a complete and active member in decision-making and choices of measures or strategies, according to its language, characteristics and particularities. This exchange of knowledge strengthens students’ training, and can awaken solidarity as well as personal skills, such as teamwork, leadership and planning^(7)^.

The process systematization using techniques or tools already validated, such as brainstorming and the PDSA cycle, provided the learning of those involved in an orderly, participatory and evaluative manner^(9)^. Its practicality also allowed scheduled actions to be executed within the proposed time. Two difficulties encountered during the use of PDSA tool stand out, which were managing the number of people involved (14 participants) and the overlapping of steps in the cycle during some meetings. Such difficulties were solved by subdividing the group, delimiting the members’ attributions and constant retakes of achieved and future steps, sending participants actions that needed to be carried out at that moment or meeting. As benefits, it is pointed out that both tools allowed the democratic exercise by the egalitarian expression of suggestions, improvements and adjustments throughout the process, generating great teamwork integration and appreciation. Moreover, an in-depth study of NVC during the two cycles made the project members themselves identify their communication failures and seek immediate implementations to resolve their interpersonal conflicts.

### Study limitations

This report presents the experience of building an educational technology to promote NVC among health professionals, carried out by members of an extension project. Despite the theoretical foundation obtained by the project members, they cannot be considered experts or judges, leaving content validation and future effectiveness tests.

### Contributions t nursing, health, and public policies

The mini NVC almanac for health professionals is a soft technology, created from a university extension project, which can help workers in their interpersonal relationships and help in the prevention and even solution of conflicts caused by communication failures in the workplace. The simple language, the chosen dynamics and the electronic availability in a public repository allow its use to reach people from different professional categories and at various levels of health care. The authors suggest that its use be prioritized, especially by health managers, in contexts where interpersonal relationship problems resulting from communication failures are identified. However, its use may not be very opportune in scenarios where serious friction between health professionals is established, or situations of persistent violence, which may require other technologies of a culture of peace, such as conflict mediation or restorative justice.

## CONCLUSION

This study aimed to report the construction of an educational technology to promote NVC for health professionals, through the use of process management tools that made it possible to operate quickly and efficiently, proving to be suitable for contexts where the creation of educational products is necessary. The proposal created, from a university extension project, illustrates the importance of extension in the education of people, its impact on society and implementation of actions that consolidate greater goods for the collective, such as a culture of peace and violence prevention.

The difficulties encountered could be quickly circumvented and did not overlap with the benefits experienced, such as teamwork and the exercise of democratic discussions by those involved. The mini NVC almanac for health professionals is a playful, creative and pleasant way for health professionals of different levels to learn about the subject. Interpersonal relationships in the health work environment can be favored by the educational technology exposed, reducing damage to those involved, witnesses and even the institution.

The final product license registration guaranteed legitimacy of its conquest by extensionists and its availability, free of charge and unrestricted, in an institutional repository that allows its use in various contexts and scenarios of action.

## Data Availability

https://repositorio.unifesp.br/handle/11600/60428, Repositório UNIFESP, Metadata.
